# Cytotoxicity of V-Prep Versus Phosphoric Acid Etchant on Oral Gingival Fibroblasts

**DOI:** 10.3390/jfb13040266

**Published:** 2022-11-28

**Authors:** Victor Ghoubril, Sylvie Changotade, Didier Lutomski, Joseph Ghoubril, Carole Chakar, Maher Abboud, Louis Hardan, Naji Kharouf, Elie Khoury

**Affiliations:** 1 Department of Orthodontics, School of Dentistry, Saint-Joseph University, Beirut 1107 2180, Lebanon; 2 Unité de Recherches Biomatériaux Innovants et Interfaces, URIT, Université Sorbonne Paris Nord—Université de Paris, 93017 Paris, France; 3 Department of Periodontology, School of Dentistry, Saint-Joseph University, Beirut 1107 2180, Lebanon; 4 Unité Environnement Génomique et Protéomique, U-EGP, Faculté des Sciences, Université Saint-Joseph, Campus des Sciences et Technologies Mar Roukos-B.P. 1514, Riad El Solh, Beirut 1107 2050, Lebanon; 5 Department of Restorative Dentistry, School of Dentistry, Saint-Joseph University, Beirut 1107 2180, Lebanon; 6 Department of Biomaterials and Bioengineering, INSERM UMR_S 1121, Biomaterials and Bioengineering, 67000 Strasbourg, France

**Keywords:** cell viability, dental acid etchants, oral mucosa, phosphoric acid, V-prep

## Abstract

The most used etchant in dental daily practice is the phosphoric acid (P.A.; 37%). However, acid etchants can induce necrosis on the oral mucosa and cause the ulceration of periodontal tissue when a rubber dam is not used. V-prep is a new practical alternative, and it has satisfactory results. It is used as a preparation before the application of a resin-modified glass ionomer composite (RMGIC) to bond the orthodontic brackets. The aim of this study was to investigate the effect of the V-prep on oral gingival fibroblasts cells by comparing the cell damage and cell viability after the use of V-prep and a conventional phosphoric acid etchant with different application times and concentrations. Therefore, Gingival fibroblasts passage 6 (GFP6) was grown and treated with an acid etchant and V-prep at three different concentrations (1:1, 1:2 and 1:10) for two different application durations (30 s and 1 min). The morphological changes, cell death and cell viability were assessed. Pyknosis, karyolysis, nucleus reversible and irreversible damages and membrane destruction were observed for both of the etchants at the higher concentrations and longer application durations. Mann–Whitney U-tests were used for the statistical analyses. The application of the V-prep for 30 s showed better values than the acid etchant did in the cell damage analysis and cell viability analysis (*p* = 0.03). V-prep at a 1:10 concentration applied for a 30 s duration can preserve the viability of gingival fibroblasts cells up to 100%. The toxicity of V-prep is equal or lower than the toxicity of the acid etchant that is commonly used in dentistry. Thus, the V-prep can be used with precautions intra-orally, and it should be applied on the enamel as a gel for 30 s only before it is rinsed and removed.

## 1. Introduction

Bracket bonding in orthodontic treatments requires a fast effective procedure with a high bond strength to resist the orthodontic forces and masticatory loads so that no bracket–enamel failure occurs [1,2,3]. Therefore, the conventional technique [4] consists of a preparation with the phosphoric acid (PA) etchant (37%) for 30 s, water rinsing, drying and bonding the brackets with a composite resin [5]. Universal adhesives with alternative techniques have been demonstrated to enhance the bond strength on the enamel and on the dentin in oral cavity restorations [6]. The drawbacks on this bonding procedure are the white spots that are encountered at the end of the treatment and the sticking on the surfaces with gingival fluid or blood excretion [7]. Another approach in the bonding procedure using a resin-modified glass ionomer cement (RMGIC) has been tested, and it has showed good results on wet surfaces, enhancing the chemical bond and releasing the fluoride during the treatment to help protect the tooth [8]. The only disadvantage was the shear bond strength of the material when it was compared to the conventional technique [9].

A previous study, which was published by the same author, has introduced a preparation procedure with V-prep before using the RMGIC. The results showed a non-significant difference in the shear bond strength (SBS) when it was compared to the PA and the composite resin [10]. To minimize the tooth damage without compromising the adhesive performance, most of the manufacturers of dental acid etchants have recommended from 15 to 30 s of use when the PA is between 32% and 40% [11]. Inadequate rinsing or remaining dental acid etchants can cause chemical burning, irritation, intra and extra-oral inflammation [12,13]. PA (37%) can lead to necrosis in the oral mucosa and ulcerative lesions of the periodontal tissue [14]. The bracket-bonding procedure for orthodontic treatments is performed without a rubber dam, while studies have demonstrated that it can protect the gingival tissue while bonding ceramic veneers [15]. V-prep has been mentioned to contain PA and sodium hypochlorite in dilution with a gel for easier manipulation. 

V-prep manipulation is similar to the conventional PA etchants as mentioned in another study [10], and should be applied for 15 to 30 s. Then, the surface should be adequately rinsed before using the RMGIC as a bonding product instead of the composite resin. Both of the etchants are applied on the bonding area of the enamel without a rubber dam protection [16]. The removal process is performed with a suction tip and high water flow, rinsing in order to dilute the etchant gel (according to the American Association of Orthodontists recommendations). Gingival fibroblasts are the first surrounding tissue to become in contact with the diluted etchant in different concentrations. Studies comparing the effect of the acid etchant on gingival fibroblasts tested three concentrations of contact, 1:1 (undiluted), 1:2 and 1:10 [17]. The toxicology and the damage caused on the oral cells have not been tested for V-prep. Cytotoxicity is essential before the use of the product in vivo [18]. Therefore, the aim of this study was to investigate the effect of V-prep on the gingival fibroblasts cells by comparing the cell damage and cell viability after the use of V-prep and a conventional phosphoric acid etchant with different application times and concentrations. 

## 2. Materials and Methods

This study was performed at URIT of Université Sorbonne Paris Nord. The study has been approved by the ethical committee of Saint-Joseph University of Beirut (USJ-2020-010).

### 2.1. Cell Cultures

Normal human gingival fibroblasts (GF), which were obtained by surgical periodontal operation, were grown in Dulbecco’s Modified Eagles Medium (Gibco BRL, Grand Island, NY, USA) supplemented with 10% fetal bovine serum and 1% penicillin/streptomycin for 6 passages. The cells were maintained in an incubator at 37 °C with an atmosphere of 5% CO_2_. The cell culture medium was changed every 3 days. The cells were detached by an enzymatic treatment with trypsin EDTA (Invitro gen, life technology) and seeded in 24-well plates at a rate of 1 × 10^5^. After 24 h, the cells were left in a culture that was untreated or treated with different dilutions (undiluted, 1:2 or 1:10) of orthophosphoric acid (Medental, Vista, CA, USA) or V-prep (concept product described in a previous article [10]) for 30 s or 1 min.

### 2.2. Giemsa Staining

To observe the morphological changes caused by orthophosphoric acid etchant and the V-prep, the cells were rinsed with PBS (Gibco, life technology, Grand Island, NY, USA), then, they were fixed according to the following procedure: absolute ethanol/PBS (50/50) at 4 °C for 5 min. Then, after 5 min of rehydration, the cells were then stained with Giemsa (Labonord, MercK, Rahway, NJ, USA) according to the supplier’s recommendations. The cells were observed at 100–250-fold magnification using an optical microscope (ZEISS AXIOPLAN, Jena, D-07740, Germany). The nucleus appeared to be deep purple, and the cytoplasm appeared be to brown or pink by light microscopy. Cell damage contains both the irreversible cell injuries, including karyorrhexis, pyknosis, karyolysis and membrane destruction, and the reversible cell injuries, including vacuole and cell swelling (enlargement).

### 2.3. Cell Death Quantification 

To quantify death cell after exposition to orthophosphoric acid etchant and V-prep, the treated cells (1 × 10^5^) with the same procedure of concentration and time for orthophosphoric acid etchant and V-prep were counted using the Beckman Coulter Vi-CELL XR (Brea, CA, USA) after Trypsin EDTA treatment to ensure the total remaining cells’ transportation into the device. The percentage of dead cells was calculated by counting the total number of cells.

### 2.4. Cytotoxicity Assay 

To identify the cytotoxic effect of dental acid etchants and V-prep on gingival fibroblast cell, a 3-(4,5-dime-thylthiazol-2-yl)-2,5-diphenyltetrazolium bromide (MTT) assay was performed. In brief, the cells (1 × 10^5^) were seeded in to 24-well plates and different concentration of orthophosphoric acid etchant (non-treated, undiluted, dilution ratios of 1:2 and 1:10, respectively) and V-prep (non-treated, undiluted, dilution ratios of 1:2 and 1:10, respectively) were applied for 30 s and 1 min (Table 1). After the cell stabilization for 24 h, MTT at 0.05 mg/mL (Sigma Aldrich, Burlington, MA, USA) was added to each well and incubated for 4 h at 37 °C. After removing the MTT solution, dimethyl sulfoxide (Merck, Rahway, NJ, USA) was added to dissolve the formazan dye crystals. The optical density was measured at a wavelength of 570 nm and a reference wavelength of 680 nm using a microplate reader (Asys UVM340, Biochrom, Cambridge, UK). The percentage of cell viability was calculated using the following formula:

(1) $$\%\mathrm{Viability}=\frac{Mean\;OD\;sample}{Mean\;OD\;untreated\;cells\;}\;\times \;100\;\;\;\;\;(OD:\;Optical\;density)$$

### 2.5. Statistical Analysis

All of the statistical analyses were performed by SPSS ver. 26.0 (IBM Corp., Armonk, NY, USA). Mann–Whitney U-tests were used to compare between the control and experimental groups. Each test was performed at least in triplicate. The results were reported as the mean ± standard deviation. A value of *p* < 0.05 was considered to be statistically significant.

## 3. Results

### 3.1. Morphological Changes Gingival Fibroblasts

**Figure 1:**
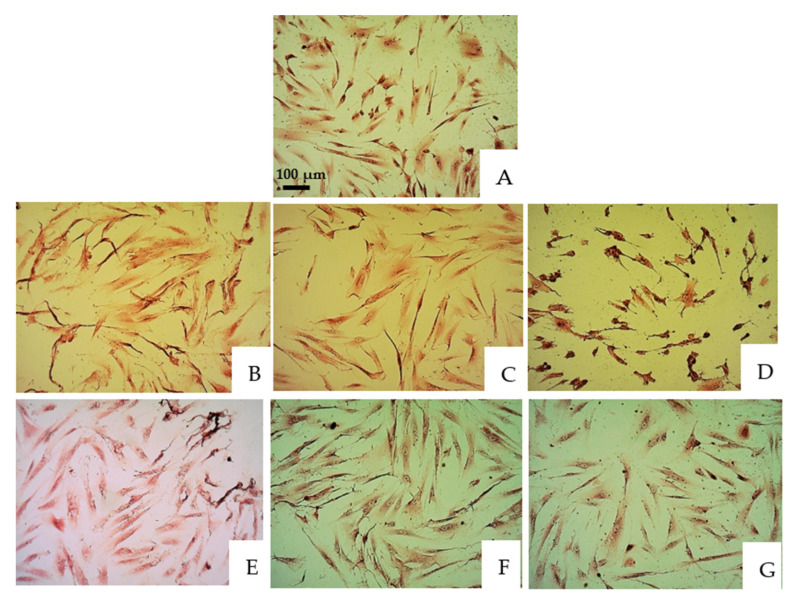
Microscope observations of oral epithelial cells stained by Giemsa under 100-fold magnification. (**A**) Control group. (**B**) V-prep, 30 s, undiluted. (**C**) V-prep, 30 s, 1:2. (**D**) V-prep, 30 s, 1:10. (**E**) Acid etchant, 30 s, undiluted. (**F**) Acid etchant, 30 s, 1:2. (**G**) Acid etchant, 30 s, 1:10.

**Figure 2:**
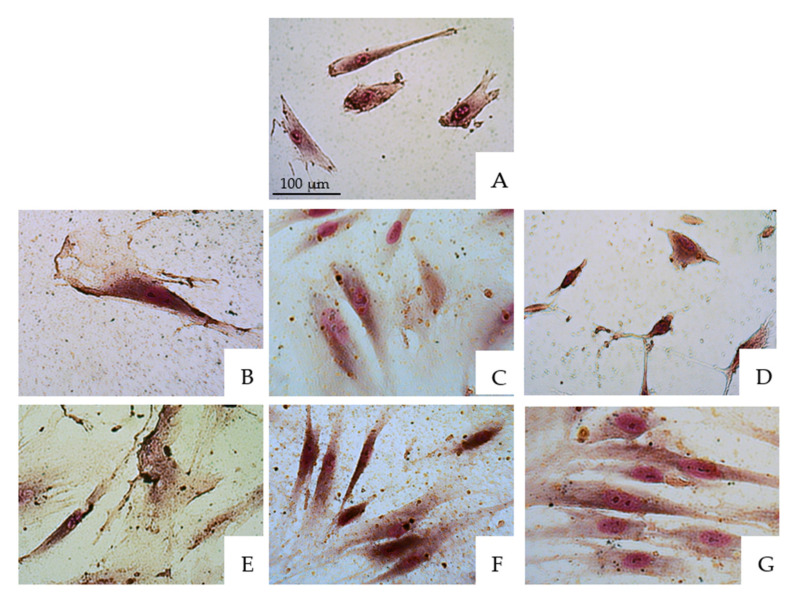
Microscope observations of oral epithelial cells stained by Giemsa under 250-fold magnification. (**A**) Control group. (**B**) V-prep, 30 s, undiluted. (**C**) V-prep, 30 s, 1:2. (**D**) V-prep, 30 s, 1:10. (**E**) Acid etchant, 30 s, undiluted. (**F**) Acid etchant, 30 s, 1:2. (**G**) Acid etchant, 30 s, 1:10.

The cells were treated with V-prep and acid etchant for 30 s and 1 min at three different concentrations, 1:1, 1:2 and 1:10. Then, Giemsa staining was performed. The same procedure was also performed on a control group in which the cells were not treated. The examinations and observations were performed using a ZEISS microscope at 100–250-fold magnification to identify the cellular changes (Figure 1 and Figure 2).

Similar pictures were taken for the microscope observations in the 30 s and 1 min application tests of the etchants, with there being no big differences. Figure 1 and Figure 2 show, respectively, the microscope observations of the oral epithelial cells stained by Giemsa under 100-fold and 250-fold magnifications with a 30 s application of the etchant. The acid etchant and V-prep caused remarkable cell damage. V-prep at a 1:10 ratio showed normal cells division when it was used for 30 s (D) when it was compared to the control group with the untreated cells (A). Pyknosis and karyolysis were observed in the low concentrations of the acid etchant (G). Cell injury in the nucleus started to be observed at a concentration of 1:2 (C,F). When the undiluted etchants were used, the percentage of cell damage was increased with an enlarged nuclear injury and subsequent membrane destruction (B,E). Cell damage contains both the irreversible cell injuries, including karyorrhexis, pyknosis, karyolysis and membrane destruction, and the reversible cell injuries, including vacuole and cell swelling. 

### 3.2. Cell Death Quantification

The percentage of dead cells was calculated by counting the total number of cells for each condition. 

**Figure 3:**
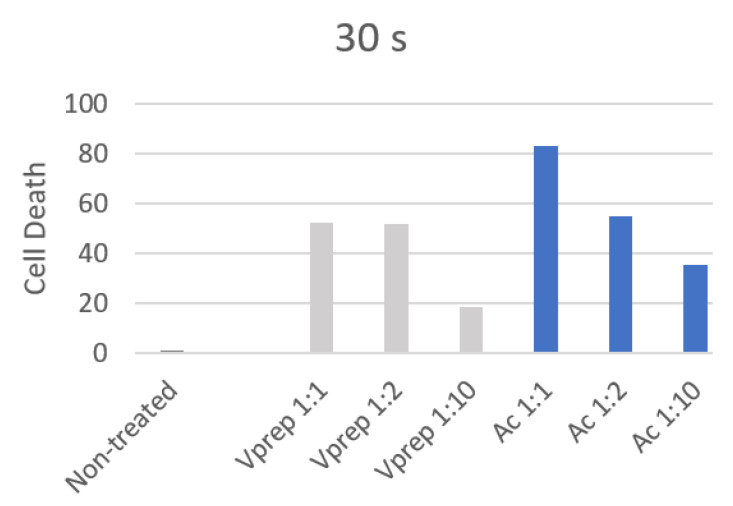
Cell death percentage after 30 s of different etchant application.

Gingival fibroblasts treated with the acid etchant for 30 s showed a significant higher death ratio (*p* < 0.001) when they were compared to the non-treated cells. The cells treated with V-prep at 1:1 (undiluted) and 1:2 for 30 s showed a significant death ratio (*p* < 0.001). Only the V-prep at a 1:10 concentration did not kill the cells significantly (*p* = 0.097) when it was used for 30 s (Table 2). The cell death rate dropped by 2.82 folds (33.58%) and by 2.35 folds (47.8%) for the V-prep and acid etchant conditions, respectively, between a 1:1 concentration and a 1:10 concentration when they were used for 30 s (Figure 3). 

**Figure 4:**
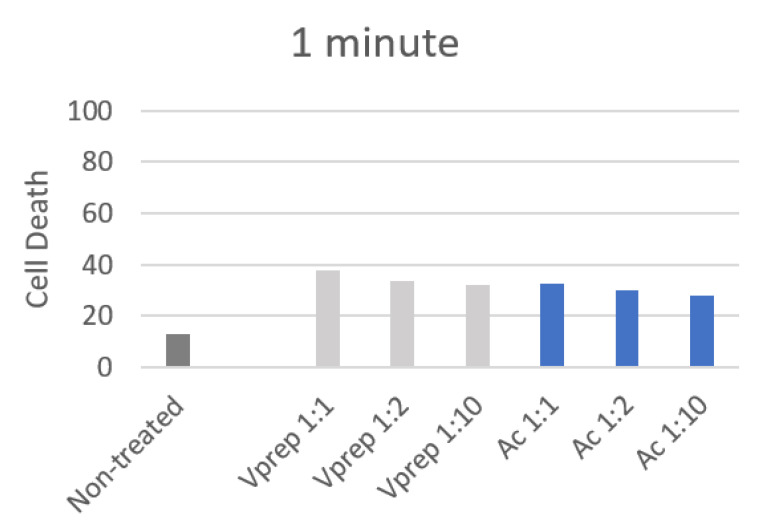
Cell death percentage after 1 min of different etchant application.

The highest value (83.25%) for the dead gingival fibroblast cells was observed for the application of undiluted acid etchant (30 s). The one minute application has showed lower values of cell death that did not exceed 37%, while the non-treated cells showed a 13% death, and a significant difference has been observed between the treated and non-treated cells for 1 min of the etchant application (*p* < 0.05). However, non-significant differences were observed between the application of acid etchant and V-prep with 1:1, 1:2 and 1:10 concentrations for 1 min (*p* = 0.8; *p* = 0.77; *p* = 0.23) (Table 3). A slight decrease in the number of dead cells of 5% and 4% were noted when the V-prep and acid etchant concentrations, respectively, were lower at 1:1 and 1:10 (Figure 4). 

### 3.3. Cell Viability Analysis

MTT assays were performed to investigate the cell viability after the application of the acid etchant and V-prep for 30 s and 1 min at different concentrations 1:1, 1:2 and 1:10. The MTT was then removed, and the cells were regrown in fresh culture for 24 h. To assess the minimum and the maximum cell viability percentages, an application of dimethyl sulfoxide (DMSO) was applied on one of the non-treated control groups (positive control), while the others were left without having any application (negative control).

All of the 30 s and 1 min cell viability values showed a decrease by a minimum of three folds when they were treated except the V-prep 1:10 treatment. The cell viability has not changed between the untreated control group and the V-prep 1:10 group when it was applied for 30 s or 1 min. 

**Figure 5:**
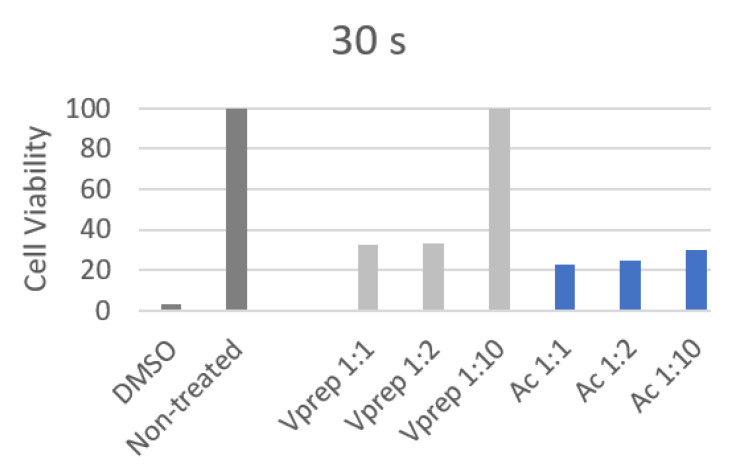
Cell viability percentage after 30 s of different etchant application.

At 30 s, the undiluted V-prep showed a higher percentage (32.8%) than the undiluted acid etchant did (22.4%) with a significant difference (*p* = 0.03) (Table 4). The V-prep at a 1:2 ratio (33%) had a cell viability value that was significantly higher than that of the acid etchant at a 1:2 ratio (25%) (*p* = 0.04) (Figure 5). 

**Figure 6:**
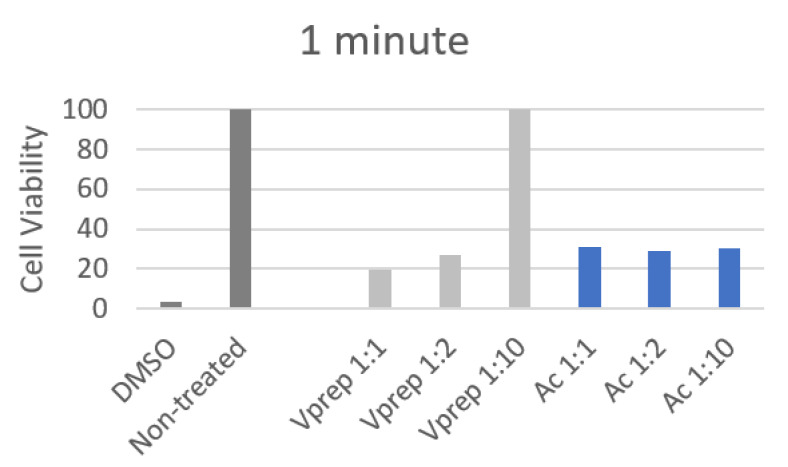
Cell viability percentage after 1 min of different etchant application.

At 1 min, the undiluted V-prep showed a lower percentage (19.5%) than the undiluted acid etchant did (31%) with a significant difference (*p* = 0.028) (Table 5). There was no significant difference between the values of the V-prep at a 1:2 ratio (27%) and the acid etchant at a 1:2 ratio (29%) (Figure 6). 

V-prep at a 1:10 ratio preserved the cells viability at a 100% rate with a very significant difference compared to the acid etchant (1:10) value (30%) (*p* < 0.001) for both of the application durations, 30 s and 1 min.

## 4. Discussion

Clinical research and studies have examined the effects of dental acid etchants on human tissues [17]. Necrosis and chemical burns were reported on the gingiva, the facial skin and the tongue [19]. Acid etchants containing 37% phosphoric acid, is commonly used in restoration treatments and in orthodontics [5]. V-prep is also a new etchant that has been tested in vitro with good shear bond strength (SBS) values when it is used with resin modified glass ionomer composite RMGIC when it is compared the values of the conventional composite bonding material prepared by an acid etchant [10]. Previous studies showed that RMGIC has many advantages over the conventional composite resin [8]. The toxicity of the V-prep preparation before bonding with RMGIC is essential in order to study the combination bonding procedure in vivo [18]. With the results of our study, our hypothesis finds that the toxicity of V-prep is similar or better when it is compared to the acid etchants that are commonly used is accepted.

In this study, the cytotoxicity tests showed similar or better effects on the gingival fibroblasts when we were comparing the V-prep to the acid etchant. Morphological changes, cell death and cell viability were investigated for acid etchant and V-prep at different concentrations (1:1, 1:2 and 1:10) and at two application durations (30 s and 1 min) on the gingival cells. First, the microscope observations showed changes between the treated and non-treated cells, such as pyknosis, karyolysis, membrane destruction, reversible and irreversible cell injuries, vacuole and cell swelling [20]. The damage was observed to be higher with the undiluted etchants without notable differences between the acid etchant and V-prep when they were used at the same concentration and for the same application time. 

Secondly, cell death was assessed in values and percentages [21]. The acid etchant showed higher values of cell death when it was compared with V-prep at the 30 s application time. The values were decreased when the etchant was diluted. The lowest value was recorded when the V-prep was applied for 30 s at a 1:10 concentration. When it was used for 1 min, all of the values were lower. The cell death quantification reports the ratio of cells that were penetrated by the colorant (trypan blue) over the total number of cells via the membrane. It does not take into consideration if the damage is reversible or irreversible which leads us to think that there being lower values at 1 min compared to 30 s are due to the gingival cells with reversible damage which occurred in the first 30 s, only. 

Thirdly, the gingival cells were analysed by viability because the cell damage is different from the potential of viability [22]. Cell damage can be reversible with slight transformations and modifications, while the cell can still be able to renew and regenerate. This leads us to say that the most accurate test is the cell viability test. The values showed that cell viability was decreased by a minimum of three folds when we used the acid etchant or V-prep. The harm was minimized when the etchant was diluted but not significantly, except for the V-prep at a 1:10 ratio. The application of the V-prep for 30 s or 1 min is harmless, and it keeps the percentage of viability at 100%.

The results of this study regarding the acid etchant application at different concentrations and durations are in complete accordance with similar studies [17,23,24]. Kim et al. found that the acid etchant can damage the vacuoles and the nucleus of the cells when it is applied for 10 s or more at a concentration of 18.5% (referring to 1:2 in our study). Accordingly, they also found that the cell viability was significantly reduced even when the acid etchant was applied for less than 30 s at a concentration of 1:10 [17]. Frob et al. found that both self-etchant adhesive and etchant-rinse adhesive methods are toxic on the normal human gingival fibroblasts [23]. Pupo et al. observed shrinkage and damage in the cells, and they identified the dental etchants to have an increased toxicity on the gingival cells [24]. All of the studies mentioned with similar results recommend in their conclusion, a wise use of the dental etchants, taking into consideration their toxicity and the manufacturer guidelines. Understanding the use of each adhesive method and its advantages over the others is necessary [25].

Practically, when it was used, the acid etchant was applied undiluted at a 1:1 ratio for 30 s before rinsing with a high flow of water for another 30 s. The next step was drying and applying the adequate bonding material in order to fix the orthodontic brackets. During the rinsing with water, the etchant is highly diluted, and the effect is reduced. The dental etchant is only used as a gel texture in order to be able to control the surface of application and limit the damage of the oral tissues [26]. However, the diluted etchant can hit the oral epithelial cells before its complete removal. Thus, better cell viability values at a 1:10 concentration are encouraging and can be a high advantage for the use of V-prep over the acid etchant.

This study encountered few limitations in the statistical analyses. The extracted values on the counting machine when we one is identifying the cell death can vary from one count to another. Therefore, the repetition of each count was required. Moreover, the action of the etchant can be concentrated differently in the same culture of cells, challenging the collection of data results. The findings of this study showed a high toxicity rate of the acid etchant application, which is in accordance with other studies results. Thus, the conventional etching technique becomes questionable. A clinical study on the comparison of the V-prep and the acid etchant can be a step forward in the evolution of bracket bonding in orthodontics. 

## 5. Conclusions

The toxicity of the V-prep showed similar or lower values when it was compared to the acid etchant. However, the acid etchant is used with recommended precautions as a preparation before bracket bonding in orthodontics. With better toxicity values, V-prep can be a substitute to acid etchants.

All of the dental etchants can damage the oral epithelial cells at all concentrations and application time durations when a rubber dam is not used. The use of any etchant should be in a gel texture to control the surface of application and limit the spread on the enamel to save the other epithelial cells from damage.

V-prep requires a gel texture to control the surface of application. It should be applied undiluted for a maximum of 30 s, and it should be well rinsed just after to reach a 10 times dilution before the contact with other oral tissues. The removal should be performed as it is conducted with the acid etchant.

## Figures and Tables

**Table 1:** 24-well plate treated for 30 s using V-prep and acid etchant in different concentrations before MTT procedure.

30 s	1	2	3	4	5	6
A	V-prep 1:1 (undiluted)			Acid etch 1:1 (undiluted)		
B	V-prep 1:2			Acid etch 1:2		
C	V-prep 1:10			Acid etch 1:10		
D	Non-treated			Non-treated		

**Table 2:** *p*-value using Mann–Whitney U test for cell death analyses after 30 s of different etchant application.

*p*-Value	Non-Treated	Vprep 1:1	Vprep 1:2	Vprep 1:10	Ac 1:1	Ac 1:2	Ac 1:10
**Non-treated**		0.000	0.000	0.097	0.000	0.000	0.000
**Vprep 1:1**	0.000		0.922	0.035	0.048	0.820	0.060
**Vprep 1:2**	0.000	0.922		0.037	0.045	0.810	0.061
**Vprep 1:10**	0.097	0.035	0.037		0.002	0.032	0.047
**Ac 1:1**	0.000	0.048	0.045	0.002		0.055	0.011
**Ac 1:2**	0.000	0.820	0.810	0.032	0.055		0.058
**Ac 1:10**	0.000	0.060	0.061	0.047	0.011	0.058	

**Table 3:** *p*-value using Mann–Whitney U test for cell death analyses after 1 min of different etchant application.

*p*-Value	Non-Treated	Vprep 1:1	Vprep 1:2	Vprep 1:10	Ac 1:1	Ac 1:2	Ac 1:10
**Non-treated**		0.000	0.012	0.012	0.012	0.014	0.015
**Vprep 1:1**	0.010		0.800	0.800	0.800	0.350	0.120
**Vprep 1:2**	0.012	0.800		0.880	0.880	0.770	0.120
**Vprep 1:10**	0.012	0.800	0.880		0.860	0.550	0.230
**Ac 1:1**	0.012	0.800	0.880	0.860		0.550	0.230
**Ac 1:2**	0.014	0.350	0.770	0.550	0.550		0.470
**Ac 1:10**	0.015	0.120	0.120	0.230	0.230	0.470	

**Table 4:** *p*-value using Mann–Whitney U test for cell viability analyses after 30 s of different etchant application.

*p*-Value	Non-Treated	Vprep 1:1	Vprep 1:2	Vprep 1:10	Ac 1:1	Ac 1:2	Ac 1:10
**Non-treated**		0.000	0.000	0.999	0.000	0.000	0.000
**Vprep 1:1**	0.000		0.880	0.000	0.030	0.035	0.034
**Vprep 1:2**	0.000	0.880		0.000	0.033	0.040	0.060
**Vprep 1:10**	0.999	0.000	0.000		0.000	0.000	0.000
**Ac 1:1**	0.000	0.030	0.033	0.000		0.072	0.053
**Ac 1:2**	0.000	0.035	0.040	0.000	0.072		0.058
**Ac 1:10**	0.000	0.034	0.060	0.000	0.053	0.058	

**Table 5:** *p*-value using Mann–Whitney U test for cell viability analyses after 1 min of different etchant application.

*p*-Value	Non-Treated	Vprep 1:1	Vprep 1:2	Vprep 1:10	Ac 1:1	Ac 1:2	Ac 1:10
**Non-treated**		0.000	0.000	0.999	0.000	0.000	0.000
**Vprep 1:1**	0.000		0.044	0.000	0.028	0.034	0.033
**Vprep 1:2**	0.000	0.044		0.000	0.062	0.070	0.068
**Vprep 1:10**	0.999	0.000	0.000		0.000	0.000	0.000
**Ac 1:1**	0.000	0.028	0.062	0.000		0.080	0.081
**Ac 1:2**	0.000	0.034	0.070	0.000	0.080		0.400
**Ac 1:10**	0.000	0.033	0.068	0.000	0.081	0.400	

## Data Availability

The data that support the findings of this study are available from the corresponding author upon reasonable request.
